# Perspective of Use of Antiviral Peptides against Influenza Virus

**DOI:** 10.3390/v7102883

**Published:** 2015-10-20

**Authors:** Sylvie Skalickova, Zbynek Heger, Ludmila Krejcova, Vladimir Pekarik, Karel Bastl, Jozef Janda, Frantisek Kostolansky, Eva Vareckova, Ondrej Zitka, Vojtech Adam, Rene Kizek

**Affiliations:** 1 Department of Chemistry and Biochemistry, Mendel University in Brno, Zemedelska 1, Brno CZ-613 00, Czech Republic; sylvie.skalickova@gmail.com (S.S.); heger@mendelu.cz (Z.H.); lidakrejcova@seznam.cz (L.K.); pekarik@node.mendelu.cz (V.P.); zitkao@seznam.cz (O.Z.); vojtech.adam@mendelu.cz (V.A.); 2 Central European Institute of Technology, Brno University of Technology, Technicka 3058/10, Brno CZ-616 00, Czech Republic; 3 Wool and Knitting Research Institute, Brno, Sujanovo namesti 3, Brno CZ-602 00, Czech Republic; bast@vup.cz; 4 Laboratory of Tumour Biology, Institute of Animal Physiology and Genetics, Academy of Sciences of the Czech Republic, Libechov CZ-277 21, Czech Republic; janda@iapg.cas.cz; 5 Institute of Virology, Slovak Academy of Sciences, Dubravska cesta 9, 84505 Bratislava, Slovak Republic; frantisek.kostolansky@savba.sk (F.K.); viruevar@savba.sk (E.V.)

**Keywords:** cationic peptides, hemagglutinin, influenza virus, membrane fusion, neuraminidase, viral replication

## Abstract

The threat of a worldwide influenza pandemic has greatly increased over the past decade with the emergence of highly virulent avian influenza strains. The increased frequency of drug-resistant influenza strains against currently available antiviral drugs requires urgent development of new strategies for antiviral therapy, too. The research in the field of therapeutic peptides began to develop extensively in the second half of the 20^th^ century. Since then, the mechanisms of action for several peptides and their antiviral prospect received large attention due to the global threat posed by viruses. Here, we discussed the therapeutic properties of peptides used in influenza treatment. Peptides with antiviral activity against influenza can be divided into three main groups. First, entry blocker peptides such as a Flupep that interact with influenza hemagglutinin, block its binding to host cells and prevent viral fusion. Second, several peptides display virucidal activity, disrupting viral envelopes, e.g., Melittin. Finally, a third set of peptides interacts with the viral polymerase complex and act as viral replication inhibitors such as PB1 derived peptides. Here, we present a review of the current literature describing the antiviral activity, mechanism and future therapeutic potential of these influenza antiviral peptides.

## 1. Introduction

Generally, some biological active peptides act in compliance with other defense mechanisms of plants or mammals [1,2,3] and can be considered as one of the first forms of “chemical” protection of eukaryotic cells against bacteria, protozoa, fungi, and viruses developed throughout the course of evolution [4,5]. These effects of natural peptides have been studied since 1970s and since then, various therapeutic activities were proposed against Gram-negative and Gram-positive bacteria [6]. The mechanisms of peptide action depend on their structure and can be enhanced by modifications of native peptides or chemically synthesized counterparts. In addition to screening of libraries of native structures, efficient peptides can be selected with commonly used phage display or *in silico* approaches [7]. Peptides can be designed to mimic or interact with conserved surface proteins and in the case of a variety of pathogens with mutagenic shift the peptide sequence could be modified to preserve therapeutic efficiency. In recent years, researchers have been exploring various methods to improve peptide synthesis technology from solid/liquid phase synthesis up to commercial scale. The economic and biological prospects have been well discussed in the strengths, weaknesses, opportunities and threats (SWOT) analysis by Fosgerau [8]. The good efficacy, safe, selectivity, and predictable metabolism are the strengths of peptide drugs production. On other hand, chemical and physical stability, prone to hydrolysis, and tendency to aggregation are the weaknesses of peptide pharmaceutics.

Influenza is highly contagious, febrile and influenza viruses cause acute respiratory disease. Influenza viruses cause illness with significant morbidity and mortality worldwide and they are considered as potential pandemic agents due to their high mutation rate, which may result in the formation of new subtypes [9,10]. The emerging threat of novel pandemic influenza strains spreading into the human population, as well as increasing resistance against conventional antiviral drug encouraged research efforts to develop new therapies against influenza viruses [11,12,13,14]. In our review, we present a comprehensive overview of peptides with therapeutic potential against specific targets of influenza viruses.

## 2. Design and Characteristics of Antiviral Peptides

Currently, the peptides are the candidate therapeutic agents that offer selectivity and specificity, low levels of side effects, and possibility of scaling up the production from mg to kg levels. On the other hand, they are predisposed to proteolytic degradation *in vivo* and are rapidly cleared from the circulation. In the case of influenza virus, the pulmonary delivery route is the simplest way to deliver therapeutic peptides to the target cells. The main advantage of this drug delivery method is to avoid enzymes of gastrointestinal tract and also to sustain large surface area for drug absorption [15]. Improved therapeutic targeting can be achieved through structural changes such as chemical modifications, cyclization or utilization of stable d-amino acids isoforms [16].

Considering the fact that viral infection is often followed by secondary bacterial infections [17], it would be highly advantageous if peptide therapy can target both the primary viral and a secondary bacterial infection. There is a possibility of complementing treatment of standard antiviral drugs with antibacterial drugs, such as neuraminidase inhibitors (oral oseltamivir and inhaled zanamivir) or M2 ion channel blockers (amantadine and rimantadine). The synergic effect and immune-modulatory role of such drug combinations have not been studied yet in protection against potential secondary bacterial infection.

Peptides can be divided into several groups based on their net charge, hydrophobicity, helicity or structure. The balance between hydrophobicity and the charge is an important marker of possible therapeutic application of peptides as well as amphipaticity and molecular mass [18,19]. Although these effects were adequately investigated in the case of antimicrobial peptides’ effects on bacteria (reviewed by Teixeira *et al.* [18]), in the case of viruses, the relation between peptide hydrophobicity and charge has not be clearly established yet. However, the toxicity effects on mammalian cells and hemolytic activity have been partially explained by Yin *et al.* [20]. Peptides with low hydrophobicity, displayed no hemolysis even at high concentrations (up to 320 μM). In striking contrast, peptides with high hydrophobicity showed hemolytic activities at all concentrations tested. This could be explained by relatively higher hydrophobicity that undergoes a structural transition in contact with bacterial-type membranes from α-helical- to β-strand-type structures compared to the corresponding peptides with lower hydrophobicity [20]. Antiviral activities of peptides have been studied to treat severe viral disease like HIV [21,22], hepatitis [23], herpes simplex [24,25], and influenza virus [26,27]. The great advantage of peptides against viruses consists in the reduced possibility of developing resistance during the treatment [28].

### Influenza Virus Replication Cycle

**Figure 1:**
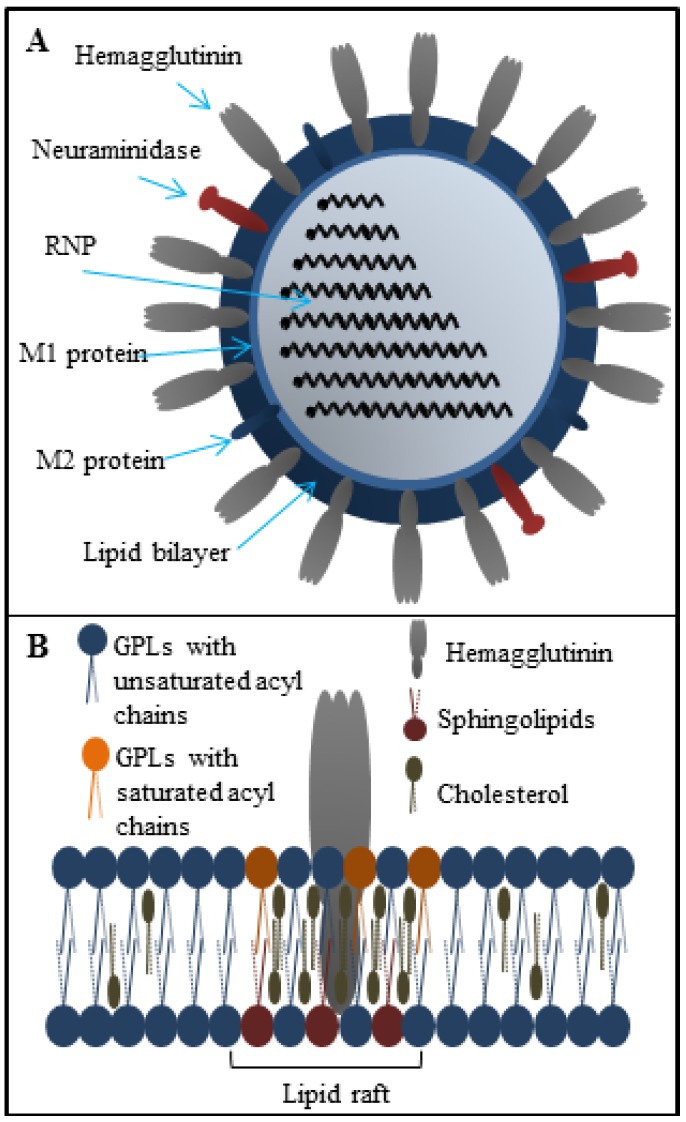
(**A**) The structure of influenza virus particle; (**B**) Structure of the lipid raft localized in the influenza lipid bilayer. The lipid rafts are composed mainly from glycolipids (GPLs), cholesterol and sphingolipids. These microdomains are responsible for the effective viral fusion.

In order to understand therapeutic potential of peptides against influenza virus, it is necessary to understand the viral replication cycle. The influenza virus (Figure 1A) is an enveloped virus of the *Orthomyxoviridae* family. The viral genome is composed of eight segments of single-stranded negative sense RNA creating a ribonucleoprotein complex (RNP) with polymerase proteins PB1, PB2, PA and nucleoprotein (NP) [29]. The life cycle starts after the attachment of virus to the host cell via viral hemagglutinin (HA). Hemagglutinin is a trimeric surface glycoprotein receptor recognizing sialic acids on the surface of host cells [30]. After virus entry through receptor-mediated endocytosis, the fusion process mediated by HA trimers is activated by low pH in late endosomes, under which HA structure is destabilized and conformationally changed. Consequently, the N-terminus of HA2 glycoprotein is exposed and inserted into the endosomal membrane resulting in the fusion of viral and endosomal membranes. Only HA molecules that are previously proteolytically cleaved into the HA1 and HA2 glycoproteins are able to mediate the fusion. The interior environment of viral particle is acidified through an ion channel formed by M2 protein, leading to the dissociation of viral matrix protein (M1) from viral ribonucleoprotein (vRNP) complex. The M1 protein is subsequently released into the host cell cytoplasm and transported to the nucleus, the site of viral RNA replication and transcription [31]. The replication and transcription of vRNA is catalyzed in the cell nucleus by viral RNA polymerase complex [32]. Influenza viral mRNA are translated by the host cell translation machinery. The newly synthetized viral proteins HA, neuraminidase (NA) and M2 proteins are transported to the plasma membrane [33]. It is generally assumed that the influenza envelope is derived from the host cell membrane, which includes lipid rafts rich in cholesterol and sphingolipids (Figure 1B). These lipid rafts serving as a platform for concentrating HA and NA for effective viral fusion and release from the host cell [34,35]. The M1 protein plays a role in the assembly process since it interacts with lipid membranes. The M2 protein, which is abundant in the infected cells, regulates the pH in endoplasmic reticulum and in transport vesicles during the HA synthesis, trimerization and its transport to the plasma membrane. The M2 protein thus ensures the correct folding of HA trimer. Neuraminidase, the second main surface glycoprotein is needed to release new assembled viron particles from the cell surface. The M2 protein, which is found in the raft periphery, appears to mediate membrane scission and particle release from the infected cells during the virus budding process [36].

## 3. Mode of Action of Various Antimicrobial Peptides with Antiviral Activity

The three main mechanisms of antiviral effects of antiviral peptides are: (i) peptides that inhibit attachment of viruses and virus-cell membrane fusion; (ii) peptides that disrupt the viral envelope; and (iii) peptides that inhibit replication of influenza virus by interacting with viral polymerase (Table 1). In this regard, the same mechanism that is described for influenza A, which is the commonly reported type of influenza in publications, have also been reported in the case of influenza B type.

### 3.1. The Peptides Inhibiting Virus Attachment and Virus-Cell Membrane Fusion

**Figure 2:**
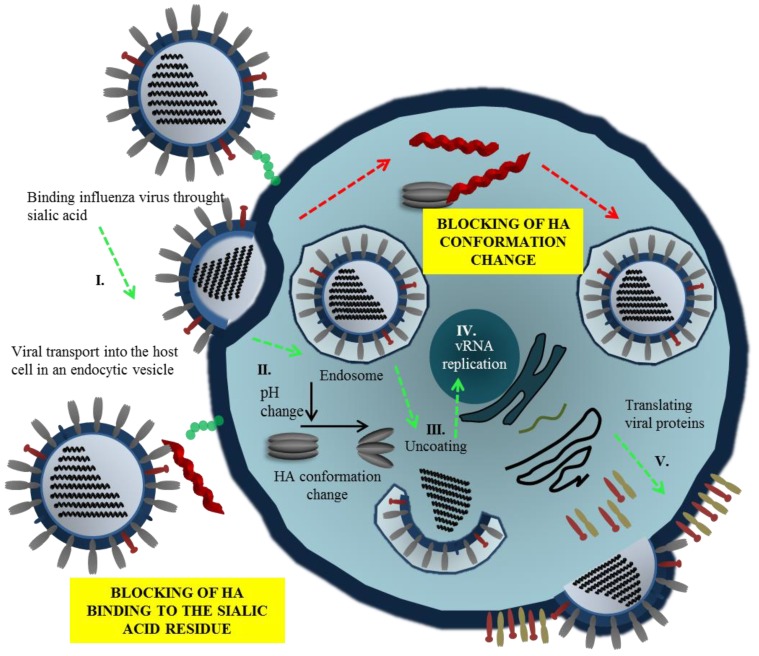
Mechanisms of inhibition of virus entry by peptides. Viral entry can be blocked via interaction of peptide with hemagglutinin (HA), commonly interacting with residue of sialic acid. This phenomenon results in the alteration of HA functions, and thus influenza virion cannot be attached to the membrane of a host cell. The second antiviral action of peptides may be carried out intracellularly due to blocking of HA conformation change that commonly leads to open of endosome and dissemination of viral genome.

Two mechanisms of inhibition of virus entry by peptides have been proposed. In the first case, the peptides compete with sialic acid (SA) binding by blocking receptor site of HA (Figure 2, Step I.). The second mechanism involves the interference with HA conformation change necessary for viral fusion (Figure 2, Step II.). Thus, the fusion of viral and endosomal membranes is blocked and release of RNA to the host cell is prevented. The viral replication and mechanisms of peptide action are shown in Figure 2.

**Table 1:** List of antiviral peptides.

**The Peptides Inhibiting Virus Attachment and Virus-Cell Membrane Fusion**							
**Peptide**	**Influenza Serotype**	**Sequence**	**Conformation**	Net Charge *	Hydrophobic Residue *	**IC_50_**	**Reference**
EB peptide	Broad spectrum	RRKKAAVALLPAVLLALLAP	linear	4	70	3 to 20 µM	[37]
Derived EB peptide	Broad spectrum	RRKKLAVLLALLA	linear	4	69	3.5 µM	[38]
P1	H9N2	NDFRSKT	linear	1	14	48 µM	[39]
P1 cyclic	H9N3	CNDFRSKTC	cyclic	1	33	71 µM	[39]
FluPep 1	H1N1	WLVFFVIFYFFR	α-helix	1	83	0.093 µM	[40]
FluPep 2	H1N1	WLVFFVIAYFAR	α-helix	1	83	0.0009 µM	[40]
FluPep 3	H1N1	WLVFFVIFYFFRRRKK	α-helix	5	62	0.00003 µM	[40]
FluPep 4	H1N1	RRKKWLVFFVIFYFFR	α-helix	5	62	0.00004 µM	[40]
FluPep 7	H1N1	RRKKIFYFFR	α-helix	5	40	0.15 µM	[40]
FluPep 8	H1N1	WLVFFVRRKK	α-helix	4	60	0.63 µM	[40]
FluPep 9	H1N1	FFVIFYRRKK	α-helix	4	50	1.48 µM	[40]
C18-s2	H1N1, H3N2	C17H35CO-ARLPRTMVHPKPAQP-NH2	-	3	33	11–15 µM	[41]
Pal L1	H5N1	C16-ARLPRTMVHPKPAQP	micelle	3	33	-	[42]
Pal M1	H5N1	C16-ARLPRTMV	micelle	2	50	-	[42]
Pal S1	H5N1	C16-ARLPR	micelle	2	40	-	[42]
Flufirvitide	Broad spectrum	-	-	-	-	-	[43]
PEP 19-2.5	H7N7, H3N2, H1N1	GCKKYRRFRWKFKGKFWFWG	α-helix	8	40	-	[44]
PEP 19-4	H7N7, H3N2, H1N1	GKKYRRFRWKFKGKWFWFG	α-helix	8	36	-	[44]
PEP 19-8D	H7N7, H3N2, H1N1	GFWFKGKWRFKKYRGGRYKKFRWKGKFWFG	α-helix	12	33	-	[44]
PEP 19-CP	H7N7, H3N2, H1N1	SSNKSTTGSGETTTA	α-helix	0	6	-	[44]
Defensins	H1N1, H3N2	ACYCRIPACIAGERRYGTCIYQGRLWAFCC	β-sheet	3	53	-	[45]
**The Peptides Disrupting Viral Envelope**							
**Peptide**	**Influenza Serotype**	**Sequence**	**Conformation**	Net Charge *	Hydrophobic Residue *	**IC_50_**	**Reference**
LF C-lobe peptide 1	H1H1, H3N2	SKHSSLDCVLRP	α-helix	1	33	4–6 pM	[46]
LF C-lobe peptide 2	H1H1, H3N2	AGDDQGLDKCVPNSKEK	α-helix	−1	23	4–7 pM	[46]
LF C-lobe peptide 3	H1H1, H3N2	NGESSADWAKN	α-helix	−1	27	22–225 pM	[46]
Mucroporin-M1	H5N1, H1N1	LFRLIKSLIKRLVSAFK	α-helix	5	58	1.03 μM	[47]
LL-37	H1N1, H3N2	LLGDFFRKSKEKIGKEFKRIVQRIKDFLRNLVPRTES	α-helix	6	35	-	[48]
**The Peptides Inhibiting Viral Replication**							
**Peptide**	**Influenza Serotype**	**Sequence**	**Conformation**	Net Charge*	Hydrophobic Residue*	**IC_50_**	**Reference**
PB1_1-25_	Broad spectrum	MDVNPTLLFLKVPAQNAISTTFPYT	α-helix	0	44	-	[49]
PB2_1-37_	H1N1, H5N1	MERIKELRDLMSWSRTREILTKTTVDHMAIIKKYTSG	α-helix	3	35	375 nM	[50]
PB1_731–757_	H5N1	ESGRIKKEEFAEIMKICSTIEELGRQK	α-helix	0	33	-	[51]
PB1_1–25_AT6Y	H1N1, H5N1	MDVNPYLLFLKVPAQ	α-helix	0	53	22–107 nM	[52]
Killer peptide	H7N1	AKVTMTCSAS	α-helix	1	50	2.6 µM	[53]
HNP-1	H3N2	CYCRIPACIAGERRYGTCIYQGRLWAFCC	β-sheet	3	51	-	[54]
Peptid 6	H1N1, H3N2	CATCEQIADSQHRSHRQMV	Zn-finger	0	36	0.7 nM	[55,56]

* Calculated by APD2: Antimicrobial Peptide Calculator and Predictor.

The entry blocker peptides are very promising prospective candidates for viral therapy applications. Jones *et al.* [37] demonstrated the use of a 20 amino acid peptide derived from signal sequence of fibroblast growth factor 4. This study confirmed the broad-spectrum activity of peptides against human, swine, and avian influenza A H1N1, H2N2, H3N2, H5N1, H5N9, and H7N3 strains and influenza B viruses. Pretreatment of mice with peptide shows 100% protection against influenza virus, demonstrated by a decrease in viral titers in the lungs of infected animals. Although postinfection treatment with peptide was not as effective as pretreatment, it should be noted that the peptide was as effective as rimantadine in protecting mice from H5N1 infection. The peptide inhibited chicken red blood cells agglutination with IC_50_ values ranging from 3 to 20 μM. These results confirmed the ability of peptide inhibit viral attachment. Furthermore, the analysis showed low cytotoxicity of the peptide for the Madin-Darby canine kidney (MDCK) cells ranging in concentrations exceeding 50 μM in medium containing 1% BSA [37]. In a subsequent study, the minimal and optimal sequence, RRKKLAVLLALLA, confers antiviral activity similar to that of EB. In addition, a newly identified peptide, RRKKVALLAVLLALLA, possessing significantly enhanced antiviral and potentially virucidal activity against influenza A was explored. The N-terminus of these peptides with characteristic sequence RRKK influenced their solubility. The results of this study showed that up to four amino acids from C-terminus and up to seven amino acids from N-terminus could be deleted while preserving the antiviral activity [37,38]. Other results indicate that peptide P1 (NDFRSKT) has the ability to interact with HA and exhibits a strong antiviral effects and negligible hemolytic activity.

FluPep is a mix of predominantly hydrophobic α-helical peptides capable of interaction with HA blocking the viral fusion. These peptides are derived from Tkip peptide, which is a mimetic for the suppressor of cytokine signaling protein, known to be active in modulating inflammatory cytokine responses and known as an effective antiviral drug against Poxviruses [57]. A variety of influenza subtypes were inhibited by FluPep in nanomolar concentrations in MDCK cells [40]. Other inhibitory peptides were identified using the Phage display library and the novel alkylated peptide with the sequence C_17_H_35_CO-ARLPRTMVHPKPAQP was retrieved. By docking simulation it was proven that the peptide was mimicking sialic acid and was recognized the by receptor-binding site in HA [41]. It seems that RLxRxMxxxK motif is crucial for the inhibitory activity, as it is homologous with highly conserved sequence within HA in many influenza strains. The amino terminal alkyl chain can play an important role in directing peptides into self-assembling micelle, stabilizing the peptide and allowing interaction with multiple binding partners. Huttl *et al.* described N-modified peptides with palmitic acid (C16-ARLPRTMVHPKPAQP, C16-ARLPRTMV, and C16-ARLPR) [42]. Due to the micelle structure of the peptides, their entropy is reduced [58,59] and affinity to HA is increased in comparison with unmodified linear peptides. Although the mechanism of the binding of the micelle peptide to the HA remains unclear, the concept has the potential of future exploits. Another peptide blocking binding of HA to sialic acid is Flufirvitide, which is currently testing in clinical trials. Besides interfering with the virus entry, it modulates the immune system by activation of production of anti-inflammatory cytokines and chemokines, increasing the activity of neutrophilic cells, and improving phagocytosis of macrophages [43]. A special group of peptides against influenza virus are cyclic delta defensins (retrocyclins), formed by coupling of N- and C-terminal domains. Their occurrence has been described in primates [43,45]. Meanwhile, previous studies have shown their ability to inhibit HIV virus by their ability to bind to HIV surface protein and the similar mechanism is supposed for influenza virus [60,61]. Another class of antiviral peptides are anti-lipopolysaccharide peptides (SALPs). The SALPs are originally based on the LPS-binding domain of Limulus anti-lipopolysaccharide-factor (LALF) and have been discovered by Gutsmann and colleagues as peptides with antimicrobial activity against Gram-positive and Gram-negative bacteria [62]. Recently, SALPs, which show antiviral activity against some enveloped viruses (HIV, HCV and HBV), have been investigated [63]. Hoffman and coworkers reported that SALPs are able to inhibit influenza virus replication of various influenza virus subtypes (H7, H3 and H1) by preventing virus attachment to host cells *in vitro* and *in vivo* by binding to N-Acetylneuraminic acids as major components of the influenza virus receptor [44].

### 3.2. The Peptides Disrupting Viral Envelope

**Figure 3:**
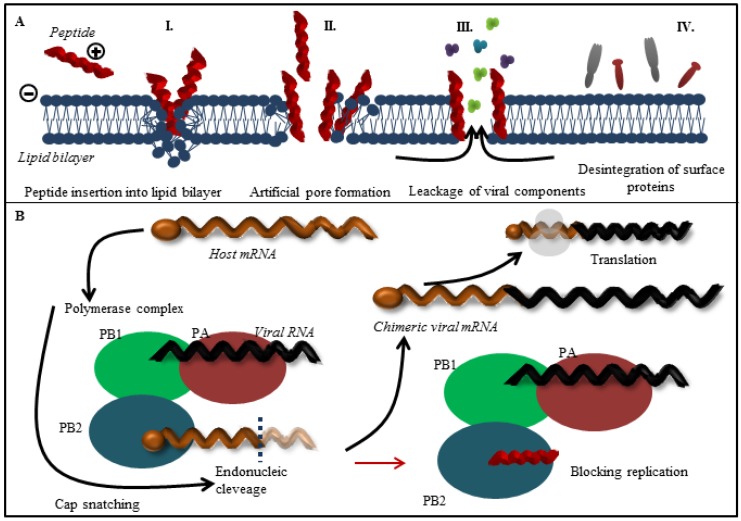
(**A**) Overall scheme of the most common interactions between antiviral peptides with an influenza virus lipid bilayer. Due to electrostatic interactions positively charged peptides are attracted by lipid bilayer with negative charge. The peptides insert into lipid bilayer (I.). The critical concentration of peptides triggers the lipid bilayer disruption. These phenomenon results in formation of artificial pores (II.) through which the low mass molecules penetrate into the capsid and contribute to the lipid bilayer destruction and leakage of viral components (III.) as well as disruption of NA and HA functions (IV.); (**B**) Scheme of function of polymerase assembly in virus replication cycle. Antiviral peptides may bind to PB2 subunit (peptides derived from PB1 subunit) and thus prevent the assembly of influenza polymerase complex via blocking of active binding site of PB2 subunit.

The viral envelope is derived from host cell membranes containing lipid rafts and is rich in sphingolipids and cholesterol [34,35], as shown in Figure 1B. These compounds provide amphipathic character and negative charge [64], which is responsible for electrostatic interactions with the positively charged cationic peptides [65,66]. Generally, peptide-membrane interactions are mediated by electrostatic interactions, while membrane disruption can be accomplished by different means. In the field of antimicrobial peptides, the mechanism of action is quite well understood (the topic is described in detailed in [67]). The viral envelope is adopted from host cell and thus does not exhibit a strong negative charge as bacterial membrane. It may seem that antiviral peptides result in less selectivity, however, several antiviral peptides that can cause the viral envelope disruption have been reported. The mechanism of antiviral action against viruses can either target the viral membrane in general or the lipid rafts rich in cholesterol. The latter case results in destabilization of viral surface proteins that are already enriched in the lipid rafts domains [68]. The common mechanisms of action are summarized in Figure 3A. Cathelicidins are human antiviral peptides that are able to disrupt viral envelope and were shown to elicit a number of host protective mechanisms such as promotion of barrier repairs, chemokine and cytokine production, modulation of dendritic cell differentiation, and T-cell polarization, as well as demonstrate potent anti-sepsis and anti-inflammatory properties [69]. One cathelicidin, namely human LL-37, is produced as a precursor of hCAP-18 that accumulates in neutrophil granules, but it may also be produced in epithelial cells as an acute response to pathogens [70]. Their mechanism of action is related to the interactions between the peptide and viral envelope by the carpet model characterized by formation of continuous layer on lipid bilayer surface resulting in membrane destabilization [71]. The potency of LL-37 against influenza virus seems to be similar to human defensins, involving direct interactions with the virus without affecting viral aggregation or inhibition of binding or uptake of virus by cells. LL-37 may be an important contributor to the initial innate defense against influenza virus [48]. Lactoferrin, widely present in various secretory fluids, is able to interact not only with the viral envelope, but also with receptors on the cell membrane of the host cells by interaction with viral hemagglutinin [72]. Therefore, it is not surprising that bovine lactoferrin was found as a possible agent with an ability to disrupt the virus envelope [73]. Three sequences derived from lactoferrin, SKHSSLDCVLRP, AGDDQGLDKCVPNSKEK, and NGESSADWAKN, inhibit influenza virus (H1H1, H3N2) activity at femtomolar concentrations [46]. To improve stability and circulation time, Balco *et al.* showed that lactoferrin could be encapsulated in liposomes without loss of activity [74]. Light, ionic strength or pH change stimuli can stimulate cargo release [75]. Thus, the liposomes could be used to preserve peptide drugs through tissues transport. Development of this approach deserves detailed attention in the future. Probably the best-known membrane disrupting peptide is melittin, a 26-amino acid peptide that forms the major component of European honeybee (*Apis mellifera*) venom [76,77]. Melittin, with a primary structure GIGAVLKVLTTGLPALISWIKRKRQQ, exhibits a variety of effects on lipid bilayer membranes, such as deformation of vesicles, formation of artificial pores, disruption, and lysis [78,79]. Currently, peptide-induced disruption of virion envelopes is vaguely understood. Two of the most discussed models of membrane disruption that are invoked for explaining the release of lipid content of the bilayer are: (i) forming of ruptures [80,81], mostly in the form of toroidal pores characterized by peptide aggregation on the lipid bilayer surface and subsequent perpendicular permeation through lipid bilayer by transmembrane potential change; and/or (ii) lipid bilayer destruction/solubilization [82,83] by the carpet mechanism, mentioned above. Lu *et al.* used real-time quartz crystal microbalance for tracing the dynamic behavior of lipid bilayers interacting with melittin. These results showed that reaching a threshold peptide concentration (typical for carpet model) followed by mass removal includes the release of lipids, probably as lipid-melittin complex, and the leakage of vesicle components [84], by disrupting the bilayer curvature leading to micellization of released lipids, is crucial [82]. Finally, the virion is destroyed by transient openings in the membrane enabling the passage of low molecular mass molecules prior to complete membrane lysis. Li and co-workers tested mucroporin and its optimized peptide variant mucroporin-M1 LFRLIKSLIKRLVSAFK and employed these peptides for antiviral action against measles, SARS-CoV and influenza H5N1 viruses [85]. Mucoporin M-1 design was based on the protein sequence of mucroporin to enhance the net positive charge of the hydrophilic side by replacing glycine and proline residues with lysine and arginine. It was found that the virucidal activity of mucroporin-M1 was notably increased, whereas the original mucroporin showed no virucidal activity with EC_50_ of 2.10 μg/mL (1.03 μM) against influenza strain H5N1. The inhibition model could be explained by direct interaction with the virus envelope, thereby decreasing the infectivity of virus. Due to this fact mucroporin-M1 analogues represents a practical tool for developing broad-spectrum antiviral agents, especially against RNA viruses [85].

### 3.3. The Peptides Inhibiting Viral Replication

Viral RNA-dependent RNA polymerase (RdRp), one of the rate-limiting enzymes for influenza virus transcription and replication [86,87], is composed of three polymerase subunits (PB1, PB2 and PA). The PB1 subunit is responsible for polymerization reaction and endonuclease cleavage [88,89], while PB2 is responsible for recognizing and binding the cap structure of host mRNAs [90,91]. The exact role of PA was recently clarified: the N-terminus PA subunit forming the domain with the endonuclease activity and PA endonuclease is responsible for cleavage of host pre-mRNA [92,93]. The RdRp is held together through noncovalent interactions. Disruption of RdRp assembly represents a remarkable opportunity to inhibit the enzyme function and virus replication (Figure 2, Step III.). For this reason, the interaction between PB1 and PA/PB2 is a promising target for design of new anti-influenza drugs (Figure 3B). Numerous authors have used PB1-derived peptides in order to interfere with polymerase function of the enzyme. Ghanem and coworkers tested kinetics of viral polymerase subunit interactions by immunoprecipitation method. PB1_1-25_ and PB1_715-740_ peptides could bind PA subunit and inhibit influenza replication cycle by interfering with the viral polymerase activity. Preferably, the PB1_715-740_ peptide binds to conserved site of influenza PA subunit, this approach represents promise tool to block most of influenza A virus strains [49]. Chase *et al.* described an ELISA-based assay to investigate peptides PB1_1-25_ and PB2_1-37_ capable of impairing polymerase complex formation [50]. The presented system does not include other factors, which could play a role in protein-protein interaction such as other binding domains, binding kinetics, and stabilization through trimer formation. This method enables to test libraries of variant small peptides. In another study, Li and coworkers used PB1_731-757_ peptide derived from influenza virus strain H5N1. The authors showed that PB1_731-757_ is capable of inhibiting viral polymerase activity and viral replication [51]. PB1 derived peptide can disrupt the interaction between the C-terminal part of PB1 (corresponding to PB1_676-757_) and the N-terminal part of PB2 (corresponding to PB2_1-40_) [51,94]. Wunderlich *et al.* investigated a peptide derived from the PA-binding domain of PB1 and found that the peptide blocked both the polymerase activity and viral spread. This work provides opportunity for developing new antivirals that specifically interfere with the polymerase complex assembly of both influenza A and B viruses [41,52]. Conti and coworkers described anti influenza effect of Killer peptide (KP); a toxin isolated from yeast with proven antimicrobial and anti-human immunodeficiency virus type 1 (HIV-1) activities [53,95]. Treatment with KP demonstrated a significant inhibitory activity on the replication of two influenza A virus strains, as evaluated by hemagglutination, hemadsorption, and plaque assays. In addition, KP demonstrated the complete inhibition of virus particle production and a marked reduction of the synthesis of viral proteins at a KP concentration of 4 µg/mL [53].

### 3.4. Other Possible Mechanisms of Influenza Virus Inhibition

Numerous studies have shown that a group of antimicrobial peptides called defensins can positively or negatively modulate infection caused by both enveloped and non-enveloped viruses [96,97]. Defensins play direct role in host against microbial infections as innate immune molecules [98] and are able to increase the activity of mucosal epithelia and inhibit the synthesis of viral RNA and proteins [54]. Salvatore and coworkers also showed that human α-defensin-1 (“human neutrophil peptide–1” (HNP-1)) effectively inhibits replication of influenza virus and synthesis of viral proteins when applied soon after infection. Further investigation indicates that viral inhibition could be caused by the modulation of protein kinase C activity in infected cells, suggesting the involvement of the PKC pathway [54]. The proposed strategy involves peptides derived from influenza matrix protein (M1). Peptide 6 was designed corresponding to a zinc finger region of the M1 sequence of influenza virus strain A/PR/8/34 (H1N1), centered around amino acids 148 to 166 [56]. The polymerase inhibitory properties of peptide 6 were evaluated on infections induced in mice by influenza A/PR/8/34 and A/Victoria/3/75 (H3N2) viruses [56]. To avoid the enzymatic breakdown of the peptide, the drug was administrated by intranasal route and was well tolerated up to a dose of 60 mg/kg/day. Based on suggested results, zinc finger peptides may provide a new class of antivirals effective against influenza virus [55].

## 4. Conclusions

Influenza spreads worldwide in yearly seasonal epidemics, and less frequent pandemics, posing a constant risk; thus, there is a need for new antiviral drugs. The current antiviral therapies have drawbacks such as side effects or selection of resistant strains. Especially, drug resistance of new viral strains compels us to devise new strategies for influenza treatment. Peptides may present the new generation of antiviral drugs with broad-spectrum activity; however, there are potential problems that need to be addressed. Peptide inhibitors of viral polymerase or viral assembly have to target intracellular processes and effective intracellular delivery of peptides still poses a great challenge. Repeated administration of the same peptide has a potential to trigger unwanted immune response. Many membrane disrupting peptides are likely to be cytotoxic through the same mechanism used to disrupt the integrity of viral envelope membrane, which is derived from host cells. Nevertheless, therapeutic applications and testing on animal models have not yet occurred for the majority of the peptides that have been studied. Nonetheless, these are not reasons to ignore the profit of antiviral peptides because careful design could curtail many of the abovementioned problems.
